# 2-Oxo-2*H*-chromen-7-yl 4-ethyl­benzoate

**DOI:** 10.1107/S2414314625010302

**Published:** 2025-11-21

**Authors:** Séverin Dri Goulizan-Bi, Hypolite Bazié, Aka Joseph N’Gouan, Abdoulaye Djandé, Rita Kakou-Yao, Claude Lecomte

**Affiliations:** a Laboratory of Material Sciences Environnement and Solar Energy Research Team: Crystallography and Molecular Physics University Félix Houphouët-Boigny 22 BP 582 Abidjan 22 Côte d’Ivoire; b Laboratory of Molecular Chemistry and Materials, Research Team: Organic Chemistry and Phytochemistry, University Joseph KI-ZERBO, 03 BP 7021, Ouagadougou 03, Burkina Faso; c CRM2, CNRS-Université de Lorraine, Vandoeuvre-lès-Nancy CEDEX BP 70239, France; University of Aberdeen, United Kingdom

**Keywords:** crystal structure, Hirshfeld surface analysis, coumarin

## Abstract

In the title compound, C_18_H_14_O_4_, the benzoate ring is oriented at a dihedral angle of 39.78 (5)° with respect to the coumarin ring system. In the crystal, the mol­ecules are linked by C—H⋯O hydrogen bonds to generate infinite (101) layers.

## Abstract 2

In the title compound, C—H⋯O hydrogen bonds generate infinite (101) sheets.

## Graphical abstract

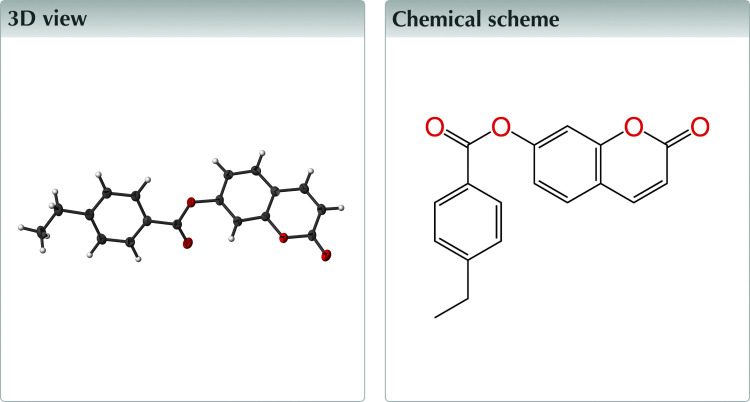

## Structure description

**Figure 1:**
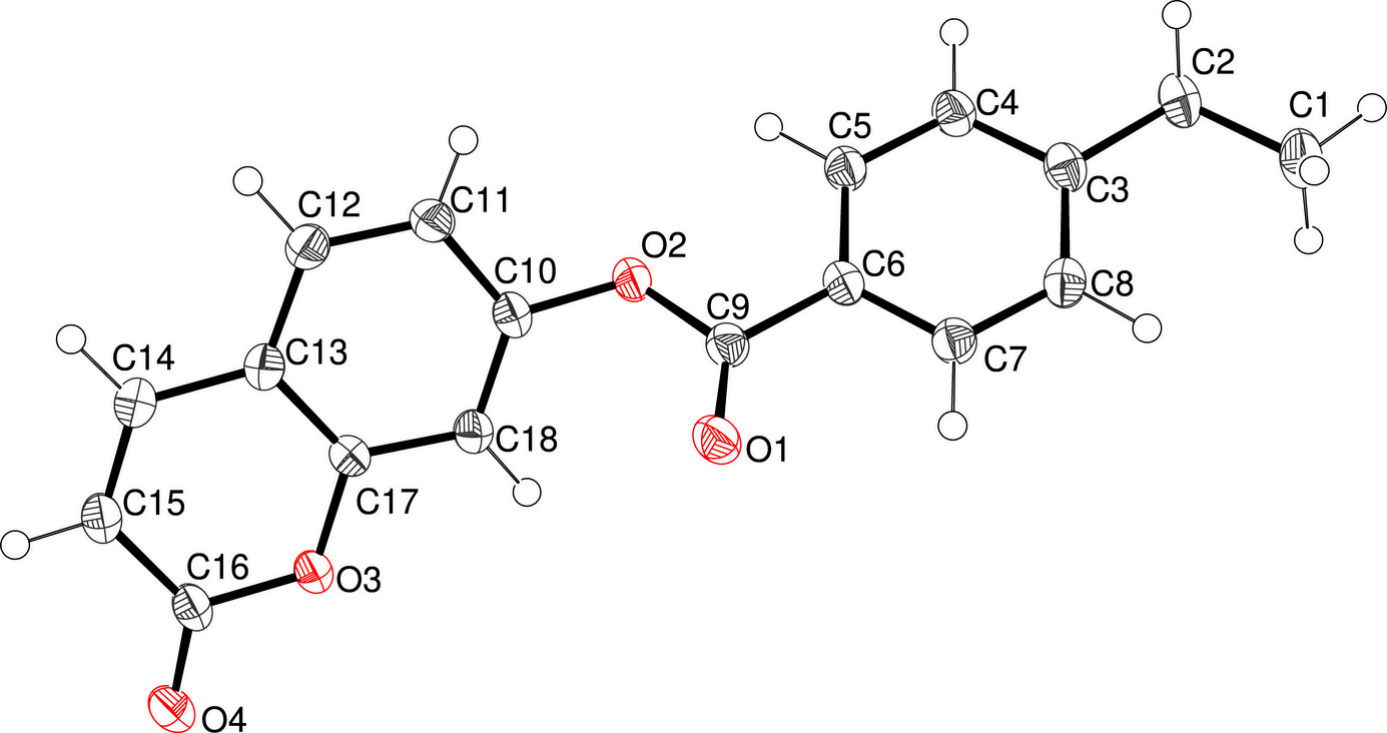
The mol­ecular structure of (**I**) with displacement ellipsoids drawn at the 50% probability level.

The title compound, C_18_H_14_O_4_ (**I**), crystallizes in the monoclinic space group *P*2_1_/*c* with one mol­ecule in the asymmetric unit (Fig. 1 ▸). The side chain is titled with respect to the chromen-2-one ring system with torsion angles C18—C10—O2—C9 = −51.22 (16)° and C11—C10—O2—C9 = 133.18 (11)°. The C10–C18/O3 coumarin ring system is almost planar (r.m.s deviation = 0.012 Å) and makes a dihedral angle with the pendant benzoate ring system of 39.78 (5)°. The C14—C15 [1.342 (2) Å] and C15—C16 [1.447 (2) Å] bond lengths are consistent with the double and single bonds in the Lewis structure of (**I**) and with those in similar coumarin derivatives (Gomes *et al.*, 2016 ▸; Ouédraogo *et al.*, 2018 ▸).

**Figure 2:**
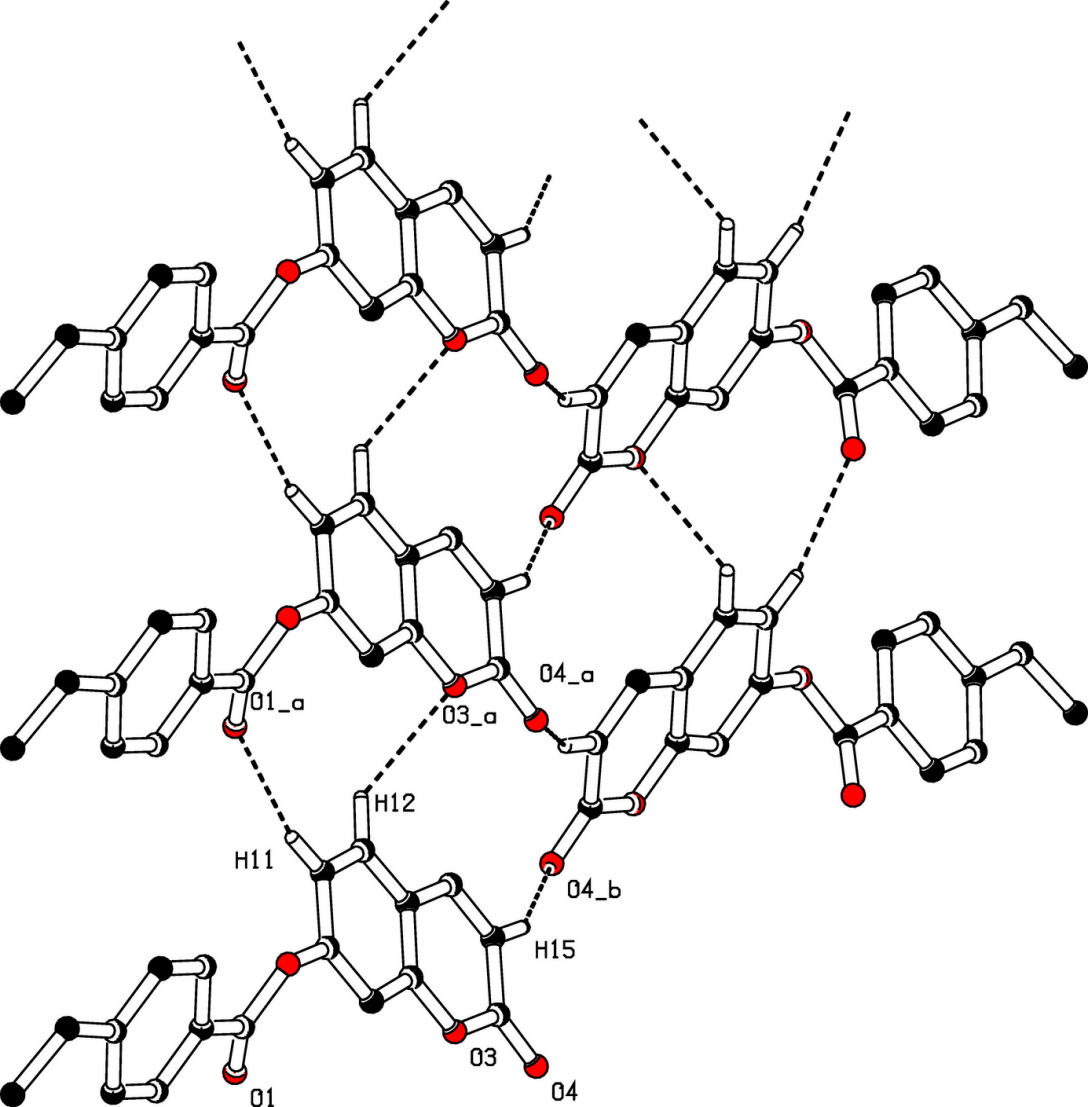
A view of the crystal packing of (**I**) showing C–H⋯O hydrogen bonds to form 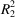
(11) and 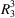
(13) loops extending parallel to the *ac* plane. H atoms not involved in the hydrogen bonds omitted.

**Figure 3:**
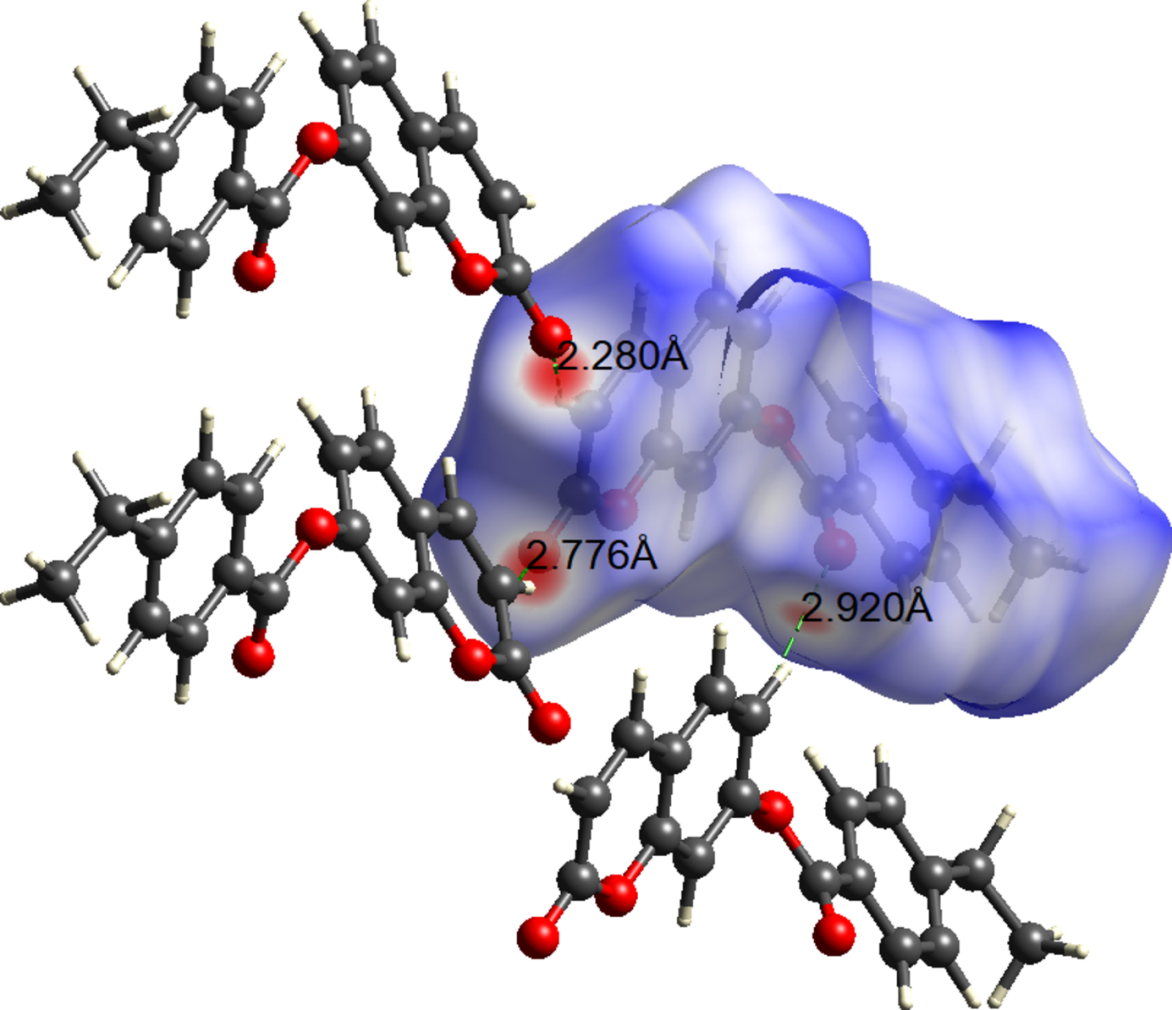
The Hirshfeld surface of (**I**) mapped over *d*_norm._ Dotted lines represent hydrogen bonds

**Figure 4:**
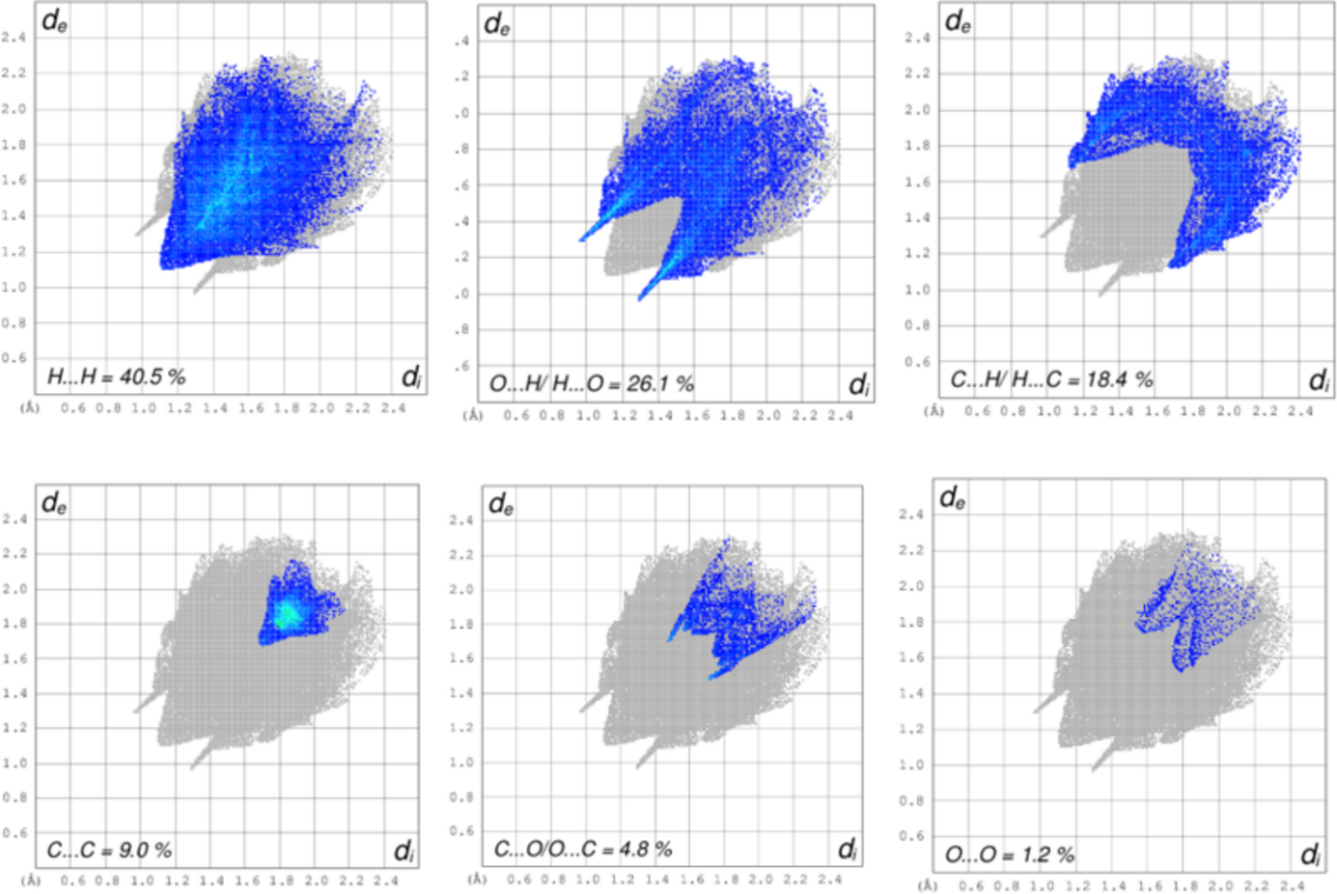
Fingerprint plots for (**I**) showing (*a*) H⋯H, (*b*) O⋯H, (*c*) C⋯H, (*d*) C⋯C, (*e*) C⋯O, (*f*) O⋯O inter­actions. The outline of the full fingerprint is shown in grey: *d*_i_ is the closest inter­nal distance from a given point on the Hirshfeld surface and *d*_e_ is the closest external contact.

In the extended structure of (**I**), the mol­ecules are connected by C—H⋯O hydrogen bonds (Table 1 ▸): the C11—H11⋯O1 and C12—H12⋯O3 bonds lead to [010] chains, which are cross-linked by the C15—H15⋯O4 bonds to generate (101) layers incorporating 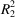
(11) and 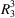
(13) loops (Fig. 2 ▸). The inter­molecular inter­actions in (**I**) were further qu­anti­fied by Hirshfeld surface (Fig. 3 ▸) analysis using *CrystalExplorer* (Spackman *et al.*, 2021 ▸): the two-dimensional fingerprint plots for (**I**) (Fig. 4 ▸) show that the greatest contributions are from H⋯H (40.5%), H⋯O/O⋯H (26.1%) C⋯H/H⋯C (18.4%) and C⋯C (9.0%) contacts.

## Synthesis and crystallization

In a 100 ml round-bottom flask equipped with a condenser, 4-ethyl­benzoyl chloride (0.95 ml, 6.2 mmol, ∼1 equiv.) was dissolved in 30 ml of tetra­hydro­furan and then were added dried tri­ethyl­amine (2.6 ml, 3 equiv.) and 7-hy­droxy­coumarin (1 g, 6.17 mmol, 1 equiv.) in small portions over 30 min. While stirring, the mixture was refluxed for 4 h and poured into 40 ml of chloro­form. The solution was acidified with dilute hydro­chloric acid until its discoloration was complete. The organic phase was extracted, concentrated in a vacuum until a slight cloudiness was obtained and cooled in an ice bath. The resulting precipitate was filtered off with suction, washed with petroleum ether and recrystallized from a chloro­form/*n*-hexane (1:3) solvent mixture resulting in a white powder of the title compound (1.15 g, 70% yield, m.p. = 407–409 K). Colorless crystals of (**I**) suitable for single-crystal X-ray diffraction analysis were then obtained by slow evaporation of an acetone solution.

## Refinement

Crystal data, data collection and structure refinement details are summarized in Table 2 ▸.

## Supplementary Material

Crystal structure: contains datablock(s) I. DOI: 10.1107/S2414314625010302/hb4544sup1.cif

Structure factors: contains datablock(s) I. DOI: 10.1107/S2414314625010302/hb4544Isup2.hkl

Supporting information file. DOI: 10.1107/S2414314625010302/hb4544Isup3.cml

CCDC reference: 2503393

Additional supporting information:  crystallographic information; 3D view; checkCIF report

## Figures and Tables

**Table 1:** Hydrogen-bond geometry (Å, °)

*D*—H⋯*A*	*D*—H	H⋯*A*	*D*⋯*A*	*D*—H⋯*A*
C11—H11⋯O1^i^	0.943 (17)	2.527 (18)	3.4535 (16)	167.8 (14)
C12—H12⋯O3^i^	0.972 (18)	2.597 (18)	3.3166 (15)	131.0 (13)
C15—H15⋯O4^ii^	0.97 (2)	2.38 (2)	3.2871 (16)	155.1 (15)

Symmetry codes: (i) 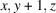; (ii) 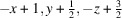.

**Table 2:** Experimental details

Crystal data	
Chemical formula	C_18_H_14_O_4_
*M*_r_	294.29
Crystal system, space group	Monoclinic, *P*2_1_/*c*
Temperature (K)	293
*a*, *b*, *c* (Å)	11.4837 (5), 6.1481 (3), 19.9255 (9)
β (°)	97.386 (2)
*V* (Å^3^)	1395.13 (11)
*Z*	4
Radiation type	Mo *K*α
μ (mm^−1^)	0.10
Crystal size (mm)	0.24 × 0.06 × 0.06

Data collection	
Diffractometer	Bruker D8 Venture
Absorption correction	Multi-scan (*SADABS*; Krause *et al.*, 2015 ▸)
*T*_min_, *T*_max_	0.712, 0.746
No. of measured, independent and observed [*I* > 2σ(*I*)] reflections	70676, 4305, 3095
*R*_int_	0.058
(sin θ/λ)_max_ (Å^−1^)	0.717

Refinement	
*R*[*F*^2^ > 2σ(*F*^2^)], *wR*(*F*^2^), *S*	0.050, 0.156, 1.06
No. of reflections	4305
No. of parameters	255
H-atom treatment	All H-atom parameters refined
Δρ_max_, Δρ_min_ (e Å^−3^)	0.22, −0.31

Computer programs: *CrysAlis PRO* (Rigaku OD, 2022 ▸), *SHELXT2018/2* (Sheldrick, 2015*a* ▸), *SHELXL2018/3* (Sheldrick, 2015*b* ▸), *PLATON* (Spek,2020 ▸) and *WinGX* (Farrugia, 2012 ▸) and *publCIF* (Westrip, 2010 ▸).

## full crystallographic data

### 2-Oxo-2H-chromen-7-yl 4-ethylbenzoate Crystal data

**Table:**

C_18_H_14_O_4_	*D*_x_ = 1.401 Mg m^−^^3^
*M**_r_* = 294.29	Melting point = 407–409 K
Monoclinic, *P*2_1_/*c*	Mo *K*α radiation, λ = 0.71073 Å
*a* = 11.4837 (5) Å	Cell parameters from 4940 reflections
*b* = 6.1481 (3) Å	θ = 5.1–61.3°
*c* = 19.9255 (9) Å	µ = 0.10 mm^−^^1^
β = 97.386 (2)°	*T* = 293 K
*V* = 1395.13 (11) Å^3^	Prism, colourless
*Z* = 4	0.24 × 0.06 × 0.06 mm
*F*(000) = 616	

### 2-Oxo-2H-chromen-7-yl 4-ethylbenzoate Data collection

**Table:**

Bruker D8 Venture diffractometer	3095 reflections with *I* > 2σ(*I*)
Radiation source: micro-focus sealed X-ray tube	*R*_int_ = 0.058
ω scans	θ_max_ = 30.7°, θ_min_ = 2.6°
Absorption correction: multi-scan (SADABS; Krause *et al.*, 2015)	*h* = −16→16
*T*_min_ = 0.712, *T*_max_ = 0.746	*k* = −8→8
70676 measured reflections	*l* = −28→28
4305 independent reflections	

### 2-Oxo-2H-chromen-7-yl 4-ethylbenzoate Refinement

**Table:**

Refinement on *F*^2^	0 restraints
Least-squares matrix: full	Hydrogen site location: difference Fourier map
*R*[*F*^2^ > 2σ(*F*^2^)] = 0.050	All H-atom parameters refined
*wR*(*F*^2^) = 0.156	*w* = 1/[σ^2^(*F*_o_^2^) + (0.0889*P*)^2^ + 0.2456*P*] where *P* = (*F*_o_^2^ + 2*F*_c_^2^)/3
*S* = 1.06	(Δ/σ)_max_ = 0.001
4305 reflections	Δρ_max_ = 0.22 e Å^−^^3^
255 parameters	Δρ_min_ = −0.31 e Å^−^^3^

### 2-Oxo-2H-chromen-7-yl 4-ethylbenzoate Special details

**Table:**

Geometry. All esds (except the esd in the dihedral angle between two l.s. planes) are estimated using the full covariance matrix. The cell esds are taken into account individually in the estimation of esds in distances, angles and torsion angles; correlations between esds in cell parameters are only used when they are defined by crystal symmetry. An approximate (isotropic) treatment of cell esds is used for estimating esds involving l.s. planes.
Refinement. The H atoms were located in difference maps and their positions and *U*_iso_ values were freely refined.

### 2-Oxo-2H-chromen-7-yl 4-ethylbenzoate Fractional atomic coordinates and isotropic or equivalent isotropic displacement parameters (Å^2^)

**Table:**

	*x*	*y*	*z*	*U*_iso_*/*U*_eq_	
O2	0.71504 (8)	0.42159 (14)	0.38731 (4)	0.0248 (2)	
O3	0.55289 (8)	0.14242 (14)	0.57921 (4)	0.0252 (2)	
O1	0.80649 (9)	0.09594 (16)	0.40536 (5)	0.0323 (2)	
O4	0.46888 (9)	0.00631 (16)	0.66357 (5)	0.0344 (2)	
C17	0.60586 (10)	0.31085 (19)	0.54905 (6)	0.0219 (2)	
C10	0.68769 (10)	0.4389 (2)	0.45336 (6)	0.0231 (2)	
C9	0.77633 (10)	0.2465 (2)	0.36850 (6)	0.0244 (2)	
C6	0.80475 (10)	0.2753 (2)	0.29849 (6)	0.0235 (2)	
C18	0.63433 (11)	0.2719 (2)	0.48430 (6)	0.0235 (2)	
C5	0.78305 (11)	0.4703 (2)	0.26297 (6)	0.0258 (2)	
C13	0.62824 (10)	0.50917 (19)	0.58229 (6)	0.0234 (2)	
C3	0.87510 (11)	0.3251 (2)	0.16904 (6)	0.0262 (3)	
C11	0.71028 (11)	0.6413 (2)	0.48389 (6)	0.0256 (2)	
C8	0.89493 (11)	0.1307 (2)	0.20483 (7)	0.0275 (3)	
C7	0.86050 (11)	0.1057 (2)	0.26900 (6)	0.0263 (2)	
C16	0.51819 (11)	0.1624 (2)	0.64297 (6)	0.0259 (3)	
C4	0.81888 (11)	0.4938 (2)	0.19946 (6)	0.0273 (3)	
C14	0.59507 (11)	0.5311 (2)	0.64935 (6)	0.0266 (3)	
C15	0.54372 (11)	0.3659 (2)	0.67835 (6)	0.0267 (3)	
C12	0.68011 (11)	0.6754 (2)	0.54797 (7)	0.0269 (3)	
C2	0.91348 (12)	0.3636 (2)	0.10002 (6)	0.0302 (3)	
C1	0.98427 (15)	0.1831 (3)	0.07278 (8)	0.0391 (3)	
H2A	0.9611 (14)	0.505 (3)	0.1025 (8)	0.034 (4)*	
H7	0.8742 (14)	−0.032 (3)	0.2945 (8)	0.035 (4)*	
H8	0.9368 (15)	0.002 (3)	0.1848 (8)	0.038 (4)*	
H4	0.8076 (14)	0.636 (3)	0.1754 (8)	0.035 (4)*	
H2B	0.8427 (15)	0.393 (3)	0.0674 (8)	0.036 (4)*	
H5	0.7432 (15)	0.596 (3)	0.2835 (8)	0.036 (4)*	
H1B	1.0623 (18)	0.151 (3)	0.1047 (10)	0.052 (5)*	
H11	0.7455 (14)	0.751 (3)	0.4603 (8)	0.033 (4)*	
H1C	0.9376 (17)	0.043 (3)	0.0665 (11)	0.059 (6)*	
H18	0.6161 (15)	0.136 (3)	0.4624 (8)	0.034 (4)*	
H1A	1.0048 (16)	0.222 (3)	0.0264 (9)	0.044 (5)*	
H12	0.6935 (15)	0.816 (3)	0.5699 (9)	0.043 (5)*	
H15	0.5194 (16)	0.377 (3)	0.7232 (10)	0.045 (5)*	
H14	0.6115 (15)	0.671 (3)	0.6738 (8)	0.037 (4)*	

### 2-Oxo-2H-chromen-7-yl 4-ethylbenzoate Atomic displacement parameters (Å^2^)

**Table:**

	*U*^11^	*U*^22^	*U*^33^	*U*^12^	*U*^13^	*U*^23^
O2	0.0296 (4)	0.0253 (4)	0.0205 (4)	0.0027 (3)	0.0076 (3)	0.0016 (3)
O3	0.0322 (5)	0.0251 (4)	0.0193 (4)	−0.0033 (3)	0.0070 (3)	−0.0004 (3)
O1	0.0364 (5)	0.0323 (5)	0.0300 (5)	0.0082 (4)	0.0115 (4)	0.0091 (4)
O4	0.0460 (6)	0.0333 (5)	0.0259 (4)	−0.0070 (4)	0.0115 (4)	0.0030 (4)
C17	0.0226 (5)	0.0231 (5)	0.0201 (5)	−0.0005 (4)	0.0033 (4)	0.0018 (4)
C10	0.0230 (5)	0.0280 (6)	0.0187 (5)	0.0018 (4)	0.0041 (4)	0.0009 (4)
C9	0.0233 (5)	0.0255 (6)	0.0252 (5)	0.0004 (4)	0.0055 (4)	0.0008 (4)
C6	0.0231 (5)	0.0272 (6)	0.0206 (5)	−0.0016 (4)	0.0042 (4)	0.0000 (4)
C18	0.0262 (6)	0.0247 (5)	0.0197 (5)	−0.0006 (4)	0.0035 (4)	−0.0013 (4)
C5	0.0279 (6)	0.0273 (6)	0.0226 (5)	0.0013 (5)	0.0051 (4)	0.0006 (4)
C13	0.0225 (5)	0.0256 (5)	0.0220 (5)	0.0017 (4)	0.0031 (4)	−0.0021 (4)
C3	0.0233 (5)	0.0335 (6)	0.0217 (5)	−0.0021 (5)	0.0031 (4)	−0.0019 (5)
C11	0.0263 (6)	0.0245 (5)	0.0270 (6)	−0.0005 (4)	0.0069 (5)	0.0017 (4)
C8	0.0258 (6)	0.0303 (6)	0.0272 (6)	0.0011 (5)	0.0061 (5)	−0.0039 (5)
C7	0.0260 (6)	0.0272 (6)	0.0260 (6)	0.0008 (5)	0.0049 (5)	0.0005 (5)
C16	0.0287 (6)	0.0310 (6)	0.0187 (5)	0.0011 (5)	0.0057 (4)	0.0018 (4)
C4	0.0296 (6)	0.0300 (6)	0.0226 (5)	0.0002 (5)	0.0044 (5)	0.0017 (5)
C14	0.0277 (6)	0.0279 (6)	0.0244 (5)	0.0010 (5)	0.0043 (4)	−0.0044 (4)
C15	0.0290 (6)	0.0311 (6)	0.0207 (5)	0.0021 (5)	0.0052 (4)	−0.0016 (4)
C12	0.0285 (6)	0.0240 (6)	0.0290 (6)	−0.0002 (4)	0.0070 (5)	−0.0027 (4)
C2	0.0282 (6)	0.0411 (7)	0.0218 (6)	−0.0004 (5)	0.0056 (5)	−0.0016 (5)
C1	0.0434 (8)	0.0451 (8)	0.0315 (7)	0.0000 (7)	0.0157 (6)	−0.0066 (6)

### 2-Oxo-2H-chromen-7-yl 4-ethylbenzoate Geometric parameters (Å, º)

**Table:**

O2—C9	1.3644 (14)	C3—C4	1.4003 (18)
O2—C10	1.3958 (13)	C3—C2	1.5157 (17)
O3—C17	1.3774 (14)	C11—C12	1.3813 (17)
O3—C16	1.3850 (14)	C11—H11	0.941 (17)
O1—C9	1.2048 (15)	C8—C7	1.3946 (17)
O4—C16	1.2126 (15)	C8—H8	1.035 (17)
C17—C18	1.3918 (15)	C7—H7	0.990 (17)
C17—C13	1.3956 (16)	C16—C15	1.4474 (18)
C10—C18	1.3807 (16)	C4—H4	0.995 (17)
C10—C11	1.3948 (17)	C14—C15	1.3417 (18)
C9—C6	1.4836 (16)	C14—H14	0.994 (17)
C6—C7	1.3925 (17)	C15—H15	0.973 (18)
C6—C5	1.3980 (17)	C12—H12	0.972 (18)
C18—H18	0.952 (17)	C2—C1	1.517 (2)
C5—C4	1.3876 (16)	C2—H2A	1.022 (17)
C5—H5	1.009 (17)	C2—H2B	0.990 (17)
C13—C12	1.4040 (17)	C1—H1B	1.05 (2)
C13—C14	1.4421 (16)	C1—H1C	1.01 (2)
C3—C8	1.3956 (18)	C1—H1A	1.011 (18)
			
C9—O2—C10	120.37 (9)	C3—C8—H8	120.9 (9)
C17—O3—C16	121.92 (9)	C6—C7—C8	120.29 (12)
O3—C17—C18	116.40 (10)	C6—C7—H7	118.4 (9)
O3—C17—C13	120.96 (10)	C8—C7—H7	121.4 (9)
C18—C17—C13	122.63 (11)	O4—C16—O3	116.24 (11)
C18—C10—C11	122.47 (11)	O4—C16—C15	126.41 (11)
C18—C10—O2	122.07 (10)	O3—C16—C15	117.35 (10)
C11—C10—O2	115.30 (10)	C5—C4—C3	121.65 (12)
O1—C9—O2	123.83 (11)	C5—C4—H4	119.7 (9)
O1—C9—C6	125.74 (11)	C3—C4—H4	118.6 (9)
O2—C9—C6	110.36 (10)	C15—C14—C13	120.76 (11)
C7—C6—C5	119.36 (11)	C15—C14—H14	120.6 (10)
C7—C6—C9	118.44 (11)	C13—C14—H14	118.6 (10)
C5—C6—C9	122.12 (11)	C14—C15—C16	121.14 (11)
C10—C18—C17	117.17 (11)	C14—C15—H15	122.6 (11)
C10—C18—H18	122.0 (10)	C16—C15—H15	116.2 (11)
C17—C18—H18	120.8 (10)	C11—C12—C13	120.82 (11)
C4—C5—C6	119.78 (11)	C11—C12—H12	120.4 (11)
C4—C5—H5	119.5 (9)	C13—C12—H12	118.8 (10)
C6—C5—H5	120.7 (9)	C3—C2—C1	116.26 (12)
C17—C13—C12	117.94 (11)	C3—C2—H2A	107.8 (9)
C17—C13—C14	117.83 (11)	C1—C2—H2A	109.2 (9)
C12—C13—C14	124.23 (11)	C3—C2—H2B	108.3 (9)
C8—C3—C4	117.86 (11)	C1—C2—H2B	109.3 (10)
C8—C3—C2	123.35 (11)	H2A—C2—H2B	105.4 (13)
C4—C3—C2	118.77 (11)	C2—C1—H1B	112.1 (10)
C12—C11—C10	118.94 (11)	C2—C1—H1C	111.5 (11)
C12—C11—H11	121.9 (10)	H1B—C1—H1C	108.3 (16)
C10—C11—H11	119.2 (10)	C2—C1—H1A	110.5 (11)
C7—C8—C3	121.06 (11)	H1B—C1—H1A	108.5 (14)
C7—C8—H8	118.1 (9)	H1C—C1—H1A	105.7 (15)
			
C16—O3—C17—C18	−178.68 (10)	O2—C10—C11—C12	176.73 (11)
C16—O3—C17—C13	0.87 (17)	C4—C3—C8—C7	−0.39 (19)
C9—O2—C10—C18	−51.22 (17)	C2—C3—C8—C7	178.31 (12)
C9—O2—C10—C11	133.19 (11)	C5—C6—C7—C8	0.37 (19)
C10—O2—C9—O1	2.72 (18)	C9—C6—C7—C8	−176.26 (11)
C10—O2—C9—C6	−174.39 (10)	C3—C8—C7—C6	0.4 (2)
O1—C9—C6—C7	7.17 (19)	C17—O3—C16—O4	177.58 (11)
O2—C9—C6—C7	−175.79 (10)	C17—O3—C16—C15	−2.29 (16)
O1—C9—C6—C5	−169.36 (13)	C6—C5—C4—C3	1.05 (19)
O2—C9—C6—C5	7.68 (16)	C8—C3—C4—C5	−0.32 (19)
C11—C10—C18—C17	−1.63 (18)	C2—C3—C4—C5	−179.08 (11)
O2—C10—C18—C17	−176.91 (10)	C17—C13—C14—C15	−0.42 (18)
O3—C17—C18—C10	−179.88 (10)	C12—C13—C14—C15	178.69 (12)
C13—C17—C18—C10	0.58 (18)	C13—C14—C15—C16	−1.07 (19)
C7—C6—C5—C4	−1.06 (19)	O4—C16—C15—C14	−177.46 (13)
C9—C6—C5—C4	175.44 (11)	O3—C16—C15—C14	2.40 (18)
O3—C17—C13—C12	−178.62 (10)	C10—C11—C12—C13	0.43 (19)
C18—C17—C13—C12	0.90 (18)	C17—C13—C12—C11	−1.41 (18)
O3—C17—C13—C14	0.54 (17)	C14—C13—C12—C11	179.49 (12)
C18—C17—C13—C14	−179.94 (11)	C8—C3—C2—C1	−4.46 (19)
C18—C10—C11—C12	1.16 (19)	C4—C3—C2—C1	174.23 (12)

### 2-Oxo-2H-chromen-7-yl 4-ethylbenzoate Hydrogen-bond geometry (Å, º)

**Table:**

*D*—H···*A*	*D*—H	H···*A*	*D*···*A*	*D*—H···*A*
C11—H11···O1^i^	0.943 (17)	2.527 (18)	3.4535 (16)	167.8 (14)
C12—H12···O3^i^	0.972 (18)	2.597 (18)	3.3166 (15)	131.0 (13)
C15—H15···O4^ii^	0.97 (2)	2.38 (2)	3.2871 (16)	155.1 (15)

Symmetry codes: (i) *x*, *y*+1, *z*; (ii) −*x*+1, *y*+1/2, −*z*+3/2.

## References

[bb1] Farrugia, L. J. (2012). *J. Appl. Cryst.* **45**, 849–854.

[bb2] Gomes, L. R., Low, J. N., Fonseca, A., Matos, M. J. & Borges, F. (2016). *Acta Cryst.* E**72**, 926–932. 10.1107/S2056989016008665 PMC4992908 27555933

[bb3] Krause, L., Herbst-Irmer, R., Sheldrick, G. M. & Stalke, D. (2015). *J. Appl. Cryst.* **48**, 3–10. 10.1107/S1600576714022985 PMC4453166 26089746

[bb4] Ouédraogo, M., Abou, A., Djandé, A., Ouari, O. & Zoueu, T. J. (2018). *Acta Cryst.* E**74**, 530–534. 10.1107/S2056989018004188 PMC5946982 29765760

[bb5] Rigaku OD (2022). *CrysAlis PRO.* Rigaku Oxford Diffraction, Yarnton, England.

[bb6] Sheldrick, G. M. (2015*a*). *Acta Cryst.* A**71**, 3–8.

[bb7] Sheldrick, G. M. (2015*b*). *Acta Cryst.* C**71**, 3–8.

[bb8] Spackman, P. R., Turner, M. J., McKinnon, J. J., Wolff, S. K., Grimwood, D. J., Jayatilaka, D. & Spackman, M. A. (2021). *J. Appl. Cryst.* **54**, 1006–1011. 10.1107/S1600576721002910 PMC8202033 34188619

[bb9] Spek, A. L. (2020). *Acta Cryst.* E**76**, 1–11. 10.1107/S2056989019016244 PMC6944088 31921444

[bb10] Westrip, S. P. (2010). *J. Appl. Cryst.* **43**, 920–925.

